# Medical Colleges

**Published:** 1919-03

**Authors:** 


### Medical Colleges.

With the return of normal conditions following the war the attendance upon the leading medical colleges of the country will be largely increased next fall. There are medical colleges and medical colleges. Physicians and others who are supervising the direction of the young medical students should exercise the greatest care in selecting the proper school for them. Beginning with the June issue of The Texas Medical Journal much attention will be paid to the subject of medical education and to the medical colleges of the country. Proper medical education is the first essential of proper medical practice. Many of the schools of the country are raising their standards. They have largely been handicapped by the depletion of their teaching staffs during the war, but most of the trained instructors are now coming back far richer in experience through the service they have been giving to their country. The American medical colleges are as good as any to be found in the world.

